# GPs’ perspectives on diagnostic tests for children: a qualitative interview study

**DOI:** 10.3399/BJGP.2023.0469

**Published:** 2024-05-14

**Authors:** Elizabeth T Thomas, Margaret Glogowska, Gail Hayward, Peter J Gill, Rafael Perera, Carl J Heneghan

**Affiliations:** Centre for Evidence-Based Medicine, Nuffield Department of Primary Care Health Sciences, University of Oxford, Oxford, UK.; Nuffield Department of Primary Care Health Sciences, University of Oxford, Oxford, UK.; Nuffield Department of Primary Care Health Sciences, University of Oxford, Oxford, UK.; Child Health Evaluative Sciences Program, Hospital for Sick Children, University of Toronto, Toronto, Canada.; Nuffield Department of Primary Care Health Sciences, University of Oxford, Oxford, UK.; Centre for Evidence-Based Medicine, Nuffield Department of Primary Care Health Sciences, University of Oxford, Oxford, UK.

**Keywords:** child, diagnostic tests, general practice, primary health care, qualitative research

## Abstract

**Background:**

Most healthcare contacts for children in the UK occur in general practice. Diagnostic tests can be beneficial in narrowing differential diagnoses; however, there is substantial variation in the use of tests for children in general practice. Unwarranted variation in testing can lead to variation in quality of care and may exacerbate health inequities. To our knowledge, no previous study has tried to understand why variation in testing exists for children in general practice.

**Aim:**

To explore GPs’ perspectives on using diagnostic tests for children in primary care and the underlying drivers of variation.

**Design and setting:**

Qualitative study in which semi-structured interviews were conducted with GPs and trainee GPs in England.

**Method:**

Interviews were conducted with 18 GPs and two trainee GPs between April and June 2023. The interviews were transcribed and analysed using reflexive thematic analysis.

**Results:**

GPs reflected that their approach to testing in children differed from their approach to testing in adults: their threshold to test was higher, and their threshold to refer to specialists was lower. GPs’ perceptions of test utility varied, including objective testing for asthma. Perceived drivers of variation in testing were intrinsic (clinician-specific) factors relating to their risk tolerance and experience; and extrinsic factors, including disease prevalence, parental concern and expectations of health care, workforce changes leading to fragmentation in care, time constraints, and differences in guidelines.

**Conclusion:**

The findings of this study identify actionable issues for clinicians, researchers, and policymakers to address gaps in education, evidence, and guidance, reduce unwarranted differences in test use, and improve the quality of health care delivered to children in general practice.

## Introduction

Children aged 0–14 years comprise one-tenth of a GP’s consultation workload in the UK.^1^ Children can pose diagnostic challenges in primary care as they may present with undifferentiated symptoms and common presentations including abdominal pain, headache, joint pains, and fatigue that may have no identifiable underlying cause.^2^ This can lead to anxiety and frustration for patients and their families, who are unable to receive treatment to alleviate their suffering, and face uncertainty about their prognosis.^3^ GPs also contend with uncertainty as there are limited guidelines or evidence-based treatments for these challenging presentations.^3^ GPs must balance the risks of over-investigation and unnecessary referrals with missing or delaying a diagnosis.

The threshold to test, treat, or refer varies among clinicians,^4^ leading to substantial variation in health care delivered to children.^5^ For example, there was a 73-fold difference in the volume of specific immunoglobulin E allergy tests ordered by UK primary care trusts in 2012.^6^ A population-based study of laboratory data showed that requests for paediatric blood tests increased by 4.2% per year from 2005 to 2019 in general practices across Oxfordshire.^7^ The reasons for this geographic and temporal variation are poorly understood. The widespread differences in test use can exacerbate health inequities in children, and both underuse and overuse of diagnostic tests lead to unintended harms for children and their families.^5^ While some degree of variation is expected as a result of differing population demographics and disease prevalence, unwarranted variation highlights tests that may be of low value.^8^ This has significant cost implications. For example, GP-requested vitamin D tests, widely considered unnecessary in asymptomatic children, cost the NHS £2 million in 2014.^9^ Beyond establishing that variation exists, it is equally important to try to understand the factors that drive variation, as it can highlight areas where clinicians, researchers, and policymakers may target efforts to improve testing.

To our knowledge, no previous study has explored why and when GPs use diagnostic tests for children in primary care. This study aims to explore GPs’ perspectives on using diagnostic tests for children in primary care to shed light on their diagnostic behaviours and better understand the factors that underpin their diagnostic decisions.

**Table:** How this fits in

Previous research has quantified paediatric diagnostic test use in general practice and identified variation in testing; however, to our knowledge, there is no research that explores paediatric testing from GPs’ perspectives. We conducted semi-structured interviews with 18 GPs and two trainee GPs. Interview participants described how their approach to testing in children differed from their approach to testing in adults, they had varied opinions about the utility of specific tests for children, and they explored the possible factors that drive variation in paediatric testing practices. Our findings highlight key actions that clinicians, researchers, and policymakers can take to reduce unwarranted variation in test use and provide equitable care for children in general practice.

## Method

### Design

We conducted a qualitative study using semi-structured telephone or online interviews with GPs in England. A qualitative approach was chosen as it allowed us to explore people’s experiences and perceptions of phenomena.

### Sampling and recruitment

GPs and GP trainees were recruited through GP groups on social media and through informal contacts in our academic primary care department. To address potential volunteer bias (GPs who test more thoughtfully and are interested in test variation may be more likely to participate), we contacted the senior chemical pathologist at the Oxford University Hospital clinical laboratories to identify GPs who requested the most tests in Oxfordshire and emailed them with our advertising material. From these initial contacts, we conducted snowball sampling to identify additional participants. Of those who agreed to participate by filling in the expression-of-interest form, a purposive sample was selected to obtain a maximum variation sample of GPs from diverse backgrounds, including sex, location, practice deprivation, practice partner status (partner, salaried GP, locum, or trainee), and years since GP qualification for non-trainees. Practice deprivation (the deprivation level of the population served by their practice) was self-reported by GPs on a scale (low, low–moderate, moderate, moderate–high, or high).

Information sheets and consent forms were emailed in advance of the interview. Verbal consent was obtained at the start of the interview; the researcher then signed the consent form on the participant’s behalf and scanned and sent the form to each participant after the interview.

### Data collection

A topic guide was developed based on available literature and expertise within the research team, with input from the patient and public involvement (PPI) advisory group. The questions explored GPs’ testing practices and their decision making related to requesting tests for children. The interview topic guide (see Supplementary Box S1 for details) was modified iteratively as the interviews progressed, based on new issues that emerged during the interviews.

The lead researcher, who is a doctoral researcher and medical doctor, and has received training in qualitative research methods, conducted the semi-structured interviews between April and June 2023. Data collection ended when the researcher, in consultation with the research team, determined that adequate information power for the emergent themes had been achieved.^10^

### Data analysis

Audio-recordings were transcribed verbatim by a transcription company, and transcripts were returned to the lead researcher to be checked and anonymised. Data were analysed using reflexive thematic analysis, supported by NVivo (version 1.6) software to organise, manage, and analyse the data. In following the process of reflexive thematic analysis recommended by Braun and Clarke,^11^ first, the lead researcher read each transcript multiple times to ensure familiarity with the data and coded all the transcripts. The researcher maintained a reflexive diary during the study design, interview, and analysis process. Initial inductive coding was developed into a coding framework, which was iteratively developed with the research team throughout the analysis. The relationship between codes was explored, and they were subsequently developed into categories and themes. These were organised into a thematic mind map to explore connections between concepts, themes, and subthemes, and the themes were refined. The findings were shared and discussed with the research team, which included PPI representatives.

### Patient and public involvement

The PPI advisory group comprised three parents of White ethnicity (two female and one male). Two representatives each had four children, and the third representative had three children.

## Results

Overall, 20 GPs participated in the interviews (interview duration 27–82 minutes), and their characteristics are summarised in Table 1. (See Supplementary Table S1 for details of the number of GPs who responded to the study advertisement and participated in the study.) Three participating GPs were identified as ‘high testers’ based on county testing numbers in 2019 (before the COVID-19 pandemic). There was a roughly equal proportion of female (55%) and male GPs (45%). There were six GP partners, seven salaried GPs, seven locum GPs, and two GP trainees. The span of GP experience ranged from 0–32 years.

**Table 1.** Self-reported characteristics of participating GPs and GP trainees, and their respective practice populations (*N* = 20)

**Characteristic**	***n* (%)**
**Sex**	
Female	11 (55)
Male	9 (45)

**GP status^a^**	
Partner	6
Salaried	7
Locum	7
Trainee	2

**Time since qualification (non-trainees), years**	
<5	6 (33)
5–14	6 (33)
≥15	6 (33)

**Geographic area^b^**	
Urban	16
Semi-rural	6
Rural	1

**Geographic region of England^c^**	
South East	6
London	5
West Midlands	2
South West	2
East	1
East Midlands	1
Yorkshire	1
North East	1
North West	1

**Deprivation level^b^**	
Low	3
Low–moderate	2
Moderate	5
Moderate–high	11
High	2

**Percentage of patients from ethnic minority background**	
<10	5
10–19	4
20–29	3
≥30	8

a *Two GPs worked in both salaried and locum roles.*

b *Totals do not add up as locums practised across different areas.*

c *One GP also worked as a locum part-time in the Shetland Islands.*

The results of the interviews are presented under the following main themes: how decisions to test differ in children compared with adults; perceived utility of tests (subthemes: general considerations and test-specific considerations); and perceived drivers of variation (subthemes: intrinsic factors and extrinsic factors).

### How decisions to test differ in children compared with adults

The sample contained GPs and trainees who described that their experiences requesting and performing tests for children differed from their adult patients. In a few cases, doctors themselves performed blood tests; in other cases, the practices had a phlebotomy service that set their own age cut-offs for doing blood tests on children. Below these thresholds, children would be referred to the local hospital to receive blood tests. The participating GPs and trainees had varying thresholds for requesting tests in children compared with in adults. Some perceived that the burden of testing on children is higher compared with adults:

*‘I mean, kids I would say I tend to have a bit of a higher threshold before doing bloods, for various reasons, especially young kids, because of the traumatic aspect of having their bloods taken. Obviously young kids can’t understand why someone’s pricking them with a needle, so there’s definitely that to take into account. I think … with an adult or someone older, you’d be a little bit more willing to* [test]*. Often they want blood tests and request that, so it definitely makes that process a little bit easier.’*

(L4, locum GP, <5 years)

Another GP mentioned the challenge of managing unexpected results:

*‘The risk that if you get an abnormal result you’re then going to have to do more testing — is there in my mind … for a child, it’s a more kind of scary and potentially unpleasant thing to have a test, whatever that is, whether it’s a blood test or an X-ray, you know, it means going to an unfamiliar environment and having something done to you.’*

(L1, locum GP, 5–14 years)

GPs also reported that children tend to present with conditions for which alternative diagnostic strategies, such as direct-to-treatment or watch-and-wait strategies, can be employed:

*‘I think we probably do less tests — or feel tests are less useful in children. They’re a self-selecting group … the vast majority of children don’t come to us with conditions that need tests. The vast majority are acute illnesses that are a clinical diagnosis with either a “direct to treatment”, or a “watch and wait” strategy with them.’*

(T1, GP trainee)

Several participants reflected that in children, the question of whether to test or not prompts them to consider whether they should refer to paediatrics; hence, their threshold to refer is lower than in adults:

*‘I think that with kids it’s different because we do have luckily a paediatric service locally who will see them fairly rapidly if we feel they need to. And I think that that in many ways defers the question of testing to someone else … A better test of whether this child needs testing is whether I think I’m prepared to send them to my secondary care colleagues and that decision is made much more easily, because I know my secondary care colleagues would see them quicker.’*

(P2, GP partner, 5–14 years)

Referral was also seen as a more appropriate route than performing the test in primary care, especially to avoid two sets of blood tests, in case GPs had missed an important test:

*‘If we’re having to do bloods on children — so for the younger children — we’re of the feeling that, probably, they need a paediatric referral — and the worry is that, if you’re doing a blood test, you really want to do the right blood test at the right time, so that you don’t want to have to bleed the child twice.’*

(P6, GP partner, 5–14 years)

### Perceived utility of tests

#### General considerations

Generally, GPs considered tests useful when needing to rule out a specific condition (such as coeliac disease), to rule in a suspected condition (such as diabetes), or to reassure parents. Tests were also seen as supportive in guiding management decisions, whether relating to treatment or supporting a referral to the specialists:

*‘The only time I do a test in primary care is if I think it will keep them out of hospital. I don’t tend to do tests in primary care if I’m going to refer anyway, because there’s no point. If secondary care will organise all the appropriate tests, there’s no point us doing tests for them to redo whatever tests it is that we do, so I only do tests in primary care if it’s something that I think I can manage in primary care, or it’s going to direct whether I need to refer or not refer.’*

(S2, salaried GP, 5–14 years)

On the other hand, GPs raised several concerns about testing in children. In addition to the physical and emotional burden of testing on children and their caregivers, GPs described the cost of testing for families who pay for transport, parking, and taking time off work. Additionally, they considered the broader cost implications for the health system, which was already facing equipment shortages, and the potential environmental cost of using these resources. From the clinician’s perspective, doing tests in children generates additional workload and can be more technically challenging, requiring more time, equipment, and staff than testing in adults.

#### Test-specific considerations

GPs had different perceptions of which tests are considered more useful for children than others. Though not strictly a diagnostic test, growth and developmental assessments were deemed appropriate as an objective measure for determining how sick a child was, and two GPs stated that these need to be used more and documented appropriately:

*‘I think in paediatrics — I mean the tests that they* [paediatricians] *would ask for are things like growth charts. I think that’s entirely appropriate. They would ask for some basic sort of stool testing before a referral to gastroenterology if you’re looking at that sort of thing. And another test which is a developmental assessment. And so I think that those are actually more important tests when it comes to chronic disease in children than frankly a* [C reactive protein]*. If a child’s growing properly and developing in the direction you’d expect them to, then that is probably a better test and I hope my specialist colleagues appreciated that test more than most biochemical lab tests.’*

(P2, GP partner, 5–14 years)

Urine and stool testing were perceived to be valuable as they are cheap and non-invasive. Faecal calprotectin was also considered a helpful test in the appropriately selected population, where the GP wanted to rule out a diagnosis of inflammatory bowel disease (IBD):

*‘I would do a faecal calprotectin because it’s cheap, it’s not invasive, and it will help me rule out things like IBD and that kind of thing.’*

(S5, salaried GP, <5 years)

There were mixed views relating to the use of objective tests for asthma, including fractional exhaled nitric oxide (FeNO) testing and spirometry, for various reasons. These included differing opinions on the clinical utility of the test, discrepancies in testing recommendations between local and national guidelines, and variation in access to the tests in general practice (especially following the COVID-19 pandemic when these tests were not performed). One of the participants felt that objective testing needed to be used more in children to prevent misdiagnosis of asthma and guide treatment decisions:

*‘I think we should be using more FeNO testing because I think we label a huge number of people asthmatic that shouldn’t be.’*

(P2, GP partner, 5–14 years)

However, other GPs believed that objective tests for asthma are generally inaccurate:

*‘I think some of it* [the reason for not using spirometry] *is an awareness of the test as being actually not that helpful and clouding the waters as well as, I mean, I don’t know, I’ve never tried to do spirometry in a young person, but I don’t know how good they would be at doing it.’*

(L1, locum GP, 5–14 years)

The role of guidelines and incentives for using diagnostic tests in childhood asthma was explored, with one GP stating that the quality measures should not necessarily be focused on performing tests, which generates more activity, but rather on improving the quality of clinical assessments to ameliorate asthma outcomes:

*‘There’s a big push from “QOF”* [Quality and Outcomes Framework] *— that we should be doing either spirometry or FeNO — or a peak flow reversibility — as an objective measure. I think their requirement is spirometry or FeNO — so that puts us in a very difficult position — but also, it’s not necessarily clinically indicated. Our priority is improving the quality of assessments — because that’s likely to be more helpful in terms of managing children’s asthma than access to tests, which are of some benefit, but that’s not the game-changer, in our view. In our view, it’s about the quality of assessments that are going on.’*

(S4, salaried GP, ≥15 years)

Aside from asthma tests, other tests that were considered less useful included vitamin D, allergy, and C reactive protein (CRP) tests. GPs generally agreed that testing for vitamin D has increased in recent years because of increased public health awareness. One GP explained the challenges related to increased requests for vitamin D tests, including the appropriate thresholds in children and whether supplementation would be a better strategy than testing:

*‘The vitamin D I find more difficult because I think that stems really from … public campaigns where people have said, for example, most people in the UK are probably deficient of vitamin D … but instead of using … vitamin D supplements, for example, they would rather want a test because obviously they have symptoms (for example, growth symptoms and tiredness symptoms) they ascribe to having low vitamin D levels. And the test itself you know … I’m not so sure we’ve figured out what the right thresholds are for vitamin D, so it’s all a good debate.’*

(S1, salaried GP, 5–14 years)

Another GP described their use of allergy tests as having changed throughout their clinical practice after recognising that they are not helpful, difficult to interpret, and costly:

*‘I never do RAST* [radioallergosorbent test] *tests — looking for allergy — and I know that some people use them a lot … They’re not easy tests to interpret at the best of times — we’re not convinced it’s a cost-effective way of assessing allergy, and so there’ll be a range of views on that, but that’s one of the things which I might have done in the past, and now I’m not doing at all.’*

(S4, salaried GP, ≥15 years)

GPs mentioned that CRP, a non-specific inflammatory marker, can be helpful in some specific contexts, for example, when referring a child with rheumatological symptoms or signs. Outside these contexts, they perceived that CRP was too vague, with an abnormal result often triggering a cascade of further tests:

*‘Very rarely would I test CRP. I think the only times I would use it is if I thought that the child had some kind of inflammatory condition, like an inflammatory arthritis or inflammatory bowel disease, where the CRP would really help with the diagnosis, because otherwise it’s an extremely vague marker. It often leads to a cascade of testing because the CRP will come back slightly high for no particular reason, so I don’t personally think it’s a very good test to use. It’s just kind of a blanket test as part of a panel of other things.’*

(S3, salaried GP, <5 years)

Some tests, such as vitamin B12 and autoimmune tests, were perceived to be inappropriate in the primary care setting and only valuable for secondary care, where specialists have the expertise to interpret results in the context of the child’s signs and symptoms, and to manage abnormal results.

One GP spoke about the challenges of navigating conversations with parents who request what they perceive to be inappropriate tests, such as nutritional deficiency tests (measuring levels of trace elements like zinc and magnesium), allergy tests, or immunoglobulin tests, alongside tests that they would consider to be appropriate when investigating a child’s presenting symptoms. They described the tension between wanting to comply with parents’ requests and just ‘ticking’ another box on the request form despite knowing the test was unlikely to be of clinical benefit:

*‘I think the harder scenario is when we’re being asked to do tests alongside ones we think are reasonable to add in other tests which we don’t think are reasonable. We don’t really, necessarily, understand the result and … I suppose nutritional deficiencies would be part of that, and I guess certain allergy tests where, actually, the allergy test isn’t that helpful — or looking at different immunoglobulins — not really that helpful*.*’*

(P6, GP partner, 5–14 years)

### Perceived drivers of variation

**Figure 1.**
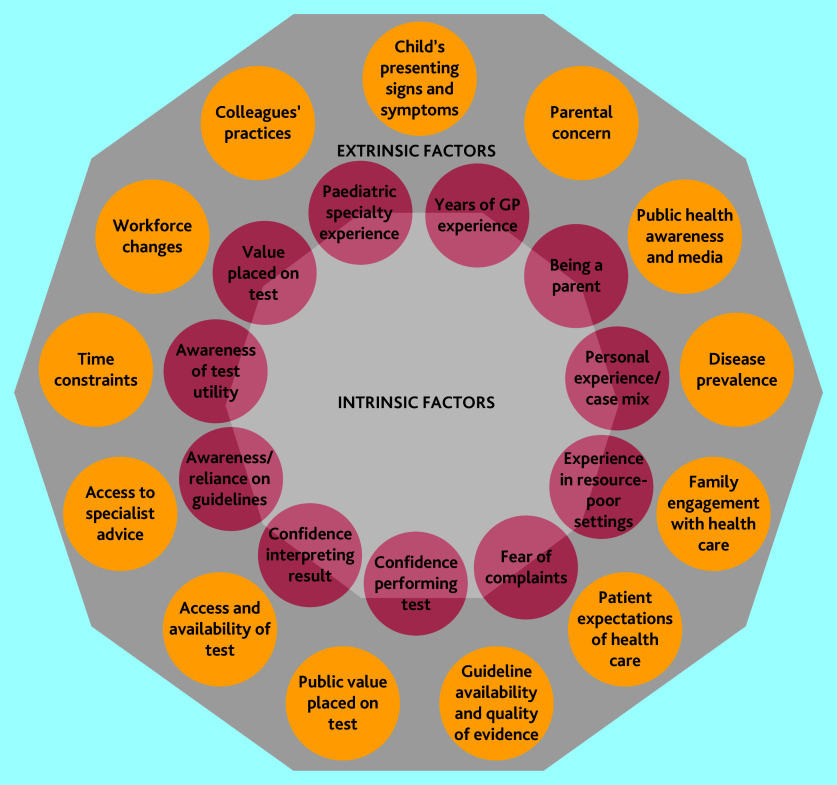
Decision-making factors that drive variation in paediatric testing

The perceived drivers of variation are summarised in Figure 1 and include intrinsic factors specific to the clinician and extrinsic factors.

#### Intrinsic factors (clinician factors)

GPs reported that variation can be partly attributed to the individual GP’s confidence in managing children and their parents. Factors contributing to confidence levels included personal paediatric specialty experience, duration of clinical experience as a GP, and experience of being a parent themselves. They reflected that personal clinical experiences may alter their behaviour; for example, a missed diagnosis in one patient may lead to searching for that diagnosis in subsequent patients. GPs who had previously worked in resource-poor settings noted that they were more judicious with their testing decisions than their colleagues.

Some GPs spoke about the technical challenges associated with doing blood tests in children, especially if there was no in-house phlebotomist. One GP talked about challenges in interpreting specific tests like electrocardiograms, X-rays, and antibody screens, and that they would only request tests for which they were confident interpreting and managing the results (see reference 1.1 in Supplementary Table S2 for supporting quote).

Several GPs spoke about the fact that variation in testing practices may be related to individual clinicians’ awareness of the utility and accuracy of tests, including the rates of false positives, and whether GPs appreciated that an ‘abnormal’ result simply refers to a statistical deviation from the population mean (see references 1.2 and 1.3 in Supplementary Table S2 for supporting quotes).

Another intrinsic factor that varied between clinicians was their awareness of and reliance on guidelines. While GPs generally knew of local guidelines and/or national guidelines, such as the National Institute for Health and Care Excellence’s Clinical Knowledge Summaries, and found them helpful, some GPs felt that guidelines could be limited in terms of providing holistic patient-centred care (see reference 1.4 in Supplementary Table S2 for supporting quote).

Additionally, GPs and trainees commented that while practising autonomously, testing practices become normative, and they adopted testing practices from their supervisors and fellow colleagues (see reference 1.5 in Supplementary Table S2 for supporting quote).

#### Extrinsic factors (non-clinician factors)

Aside from the severity of the child’s presenting condition, the most frequently cited factor that influenced the decision to test was the parent’s degree of concern and the perceived likelihood of the result reassuring the parent (see reference 1.6 in Supplementary Table S2 for supporting quote). GPs reported that differences in the population influenced their testing behaviours. Some ethnic minority groups are known to have a higher risk of having specific conditions, prompting the GP to consider testing for these conditions, such as vitamin D tests for children of Bangladeshi or African origin. One GP mentioned that for Somali patients, they were more likely to accommodate parent requests for tests to forge positive therapeutic relationships and trust between families and healthcare providers (see reference 1.7 in Supplementary Table S2 for supporting quote). Similarly, GPs felt they had to test opportunistically in patient groups who are more disadvantaged and presented to their GP less frequently (see reference 1.8 in Supplementary Table S2 for supporting quote).

Conversely, GPs perceived that patients from some ethnic backgrounds may hold different expectations of health care based on their experience of health systems in their country, and view the GP as a barrier to receiving tests and specialist care (see reference 1.9 in Supplementary Table S2 for supporting quote). Perceived public beliefs of tests being of high value and providing technically accurate results also increases parental requests for testing (see reference 1.10 in Supplementary Table S2 for supporting quote).

Other external factors of testing variation included funding, accessibility, availability of tests, and whether there was good access to specialist care, in which case testing would be deferred to their paediatrician colleagues (see references 1.11 and 1.12 in Supplementary Table S2 for supporting quotes).

GPs also believed that unnecessary testing might result from time constraints, curtailing GPs’ abilities to perform a focused history and physical examination to narrow down the differential diagnosis, and causing them instead to rely on tests. A lack of time also limits discussions relating to the benefits and harms of testing and its uncertainties (see reference 1.13 in Supplementary Table S2 for supporting quote).

Limited time and working in a pressured environment may also lead to testing that might not be appropriate when the boundaries of adult care become blurred with paediatric care. This might occur in the case of vitamin B12 testing, for example, which GPs believe is usually unnecessary for children in the primary care setting (see reference 1.14 in Supplementary Table S2 for supporting quote).

Workforce changes were highlighted as potential contributors to unnecessary testing. One GP mentioned that erosions in the continuity of care, with fewer doctors in substantive roles and more locums, may lead to more testing, with clinicians trying to be as ‘thorough’ as possible and ‘paint a complete picture’ by using tests (see reference 1.15 in Supplementary Table S2 for supporting quote).

Finally, GPs explained that guidelines influenced their decisions to test but that guideline recommendations varied by local area, sometimes differed from national guidance, and did not always consider local systems and test accessibility (see reference 1.16 in Supplementary Table S2 for supporting quote). Guidelines were also criticised for sometimes misrepresenting different recommendations with mixed quality of evidence uniformly, and for being less applicable to primary care (see reference 1.17 in Supplementary Table S2 for supporting quote). Some GPs were unaware whether there were local guidelines for children in primary care, whereas some GPs were overwhelmed by the sheer number of guidelines to stay abreast of. In certain areas, local paediatricians and/or GPs had developed many local guidelines related to caring for children in the community. Variation in guidelines may therefore be a factor that contributes to testing variation.

## Discussion

### Summary

Testing in children requires specific considerations and may carry substantial consequences. This study found that GPs’ diagnostic decision making for children differed from their decision making for adults: their threshold to request a test was higher for children, and their threshold to refer to specialty care was lower for children. Some tests, like faecal calprotectin and urine tests, were considered useful, while other tests, such as CRP, vitamin D, and spirometry, were seen to be of less value in children. Multiple factors were suggested to account for variation between clinicians and practices in the rates of test use in children, including disease prevalence, changes in the workforce leading to fragmentation in care, individual clinicians’ risk tolerance, perceived increases in parental requests, differing patient expectations of health care, local system factors, and problems with guidelines.

### Strengths and limitations

This study is the first, to our knowledge, to explore GPs’ perspectives on how, when, and why they choose to employ (or not employ) diagnostic tests in children. Participants worked in various geographic settings throughout England (and elsewhere) in practices that served diverse population groups. Rigorous and systematic methods were used to collect and analyse the data. To ensure the trustworthiness of the findings, one of the co-authors, a senior qualitative researcher, checked the transcripts, codes, and coding framework. The analysis was supported by practising GPs in the authorship team who supported the credibility of the identified themes and subthemes.

Recruitment for this study was advertised through GP groups, including our academic primary care department and the overdiagnosis working group of the Royal College of General Practitioners, which we identified as an important source of bias in our study sample as these groups are likely to have an interest in reducing unnecessary testing. To address this, we expanded our study recruitment to general GP groups on social media, where a substantial proportion (30%) of interview participants were subsequently recruited. We also included GPs identified as high testers (a further 15% of the participant sample) to address this volunteer bias and add to the range of perspectives captured in our interview sample.

This study interviewed GPs to gather their recollections and perspectives on healthcare encounters with children and their families. A limitation of the study was that it was restricted to GPs’ perspectives. These do not account for all primary care healthcare personnel who request tests, including advanced nurse practitioners, specialist and practice nurses, pharmacists, and paramedics.^12^

### Comparison with existing literature

To our knowledge, no studies have explored the use of diagnostic tests for children in primary care. This is likely due to the relative infrequency of testing for children compared with adults, where over-testing has been identified as a major problem and source of waste in the NHS.^13^ A previous qualitative interview study explored and compared GPs’ and adult patients’ expectations, experiences, and understanding of tests in primary care.^14^ The study found that in most cases, patients rarely requested tests; however, they viewed them as a positive step in moving forward with the consultation and affirmation that their GP took their concerns seriously. The decisions to request a test were led mainly by doctors, with no examples of shared decision making or information sharing. In contrast, in our study many of the GPs interviewed felt that parental request was one of the most common reasons for them to order a test, with parents sometimes entering consultations with the expectation that the outcome would be their child receiving a test. In the previous study carried out by Watson *et al*,^14^ GPs believed that time and workload pressures presented barriers to a shared understanding of testing and uncertainty, similar to the findings of our study.

Gill and colleagues^15^ conducted an interview study to examine GPs’ views on quality markers for children in primary care. Participating GPs, who all practised in the Thames Valley region, shared the concerns of the GPs in our study that a lack of standardised training influenced decision making and quality of care. Similarly, GPs in their study also felt that the principles of decision making would need to be modified to meet the needs of vulnerable children at risk, such as those with chronic illnesses.^15^

Another qualitative study in UK primary care explored GPs’ and nurse practitioners’ approaches to inflammatory marker testing in primary care.^16^ The authors identified issues that were also consistent with our findings, including ambivalence relating to the appropriate use of inflammatory markers and the tension between not wanting to miss a diagnosis and being wary of unexpected results that are borderline or inconclusive, which can lead to testing cascades. Participants in our sample also specifically reported a lack of public and clinician awareness about test utility. This is supported by systematic reviews by Hoffmann and colleagues, which found that patients^17^ and clinicians^18^ overestimate the benefits of tests/screening and underestimate the harms.

It was interesting to elicit GPs’ opinions about the perceived utility of tests in our study. Some participants identified faecal calprotectin as a helpful test in primary care. This is supported by a diagnostic accuracy study of faecal calprotectin in UK general practice,^19^ which found that the test had 100% sensitivity and 91% specificity, distinguishing between IBD and functional gut disorder in children aged between 4 and 18 years, resulting in fewer unnecessary referrals and diagnostic tests in secondary care.

On the other hand, GPs expressed mixed views about using FeNO tests to diagnose childhood asthma. Two diagnostic accuracy meta-analyses examining the diagnostic accuracy of FeNO testing in diagnosing asthma found that it performed moderately well in terms of diagnostic accuracy, with estimates of sensitivity of 0.80 and specificity ranging from 0.64 to 0.81 using a FeNO threshold of <20 parts per million.^20^^,^^21^ The availability of the test varied among GPs, and they reported there was conflicting guidance on whether to use objective asthma testing or rely on clinical features alone.

### Implications for research and practice

The use of tests varies by clinician and practice, and trends have changed substantially over time. Variation in testing practices may appropriately reflect differences in patient demographics, disease prevalence, and patient preferences; however, unwarranted variation can also highlight tests that are not clinically indicated or evidence-based. Such variation can lead to poor health outcomes and worsen health inequities.^6^ The GPs in our interview sample described many factors that underpin testing variation, and the specific drivers differ according to the child, parent, test, clinician, and local health service. This study highlights the following areas for clinicians and policymakers to target to improve testing and reduce unwarranted variation:

- emphasising clinical history taking and examination for a more accurate pre-test probability (the likelihood of a child having the pre-specified condition before receiving the diagnostic test), and how it differs in the context of ethnic variation, such as vitamin D;
- developing specific strategies or tools for clinicians to communicate uncertainty in diagnosis and test utility to a child and their parent/guardian;
- enhancing GPs’ awareness and education about the utility of specific tests and different reference ranges for children;
- increasing paediatric exposure in general practice training to improve clinicians’ confidence in managing childhood presentations;
- improving GPs’ awareness of the diagnostic guidelines that exist for children;
- refining local guidance to account for the access and availability of tests and reflect the realities of working in primary care; and
- improving the quality of the evidence base for diagnostics in children.

Future research should explore the perspectives of children and parents of children who have undergone tests in primary care to gain a more holistic understanding of the experience of diagnostic tests in this setting. The generalisability of some of the findings of this study could be studied using a data-driven approach, for example, by assessing whether less experienced clinicians test more frequently than more experienced clinicians. It is also essential to identify which tests are subject to the largest variation in use, to improve clinicians’ awareness and diagnostic guidance for these tests, and ensure that children do not undergo unnecessary testing.

## References

[1] Hobbs FDR, Bankhead C, Mukhtar T (2016). Clinical workload in UK primary care: a retrospective analysis of 100 million consultations in England, 2007–14. Lancet.

[2] Geist R, Weinstein M, Walker L, Campo JV (2008). Medically unexplained symptoms in young people: the doctor’s dilemma. Paediatr Child Health.

[3] Stone L (2015). Managing medically unexplained illness in general practice. Aust Fam Physician.

[4] Foot C, Naylor C, Imison C (2010). The quality of GP diagnosis and referral.

[5] Hiscock H, Perera P, Mclean K (2016). Variation in paediatric clinical practice: a review of care in inpatient, outpatient and emergency department settings. J Paediatr Child Health.

[6] Public Health England (PHE) (2017). The 2nd atlas of variation in NHS diagnostic services in England.

[7] Thomas ET, Withrow DR, Shine B (2024). Trends in diagnostic tests ordered for children: a retrospective analysis of 1.7 million laboratory test requests in Oxfordshire, UK from 2005 to 2019. Arch Dis Child.

[8] NHS RightCare (2012). NHS atlas of variation in healthcare for children and young people.

[9] Basatemur E, Hunter R, Horsfall L (2017). Costs of vitamin D testing and prescribing among children in primary care. Eur J Pediatr.

[10] Malterud K, Siersma VD, Guassora AD (2016). Sample size in qualitative interview studies: guided by information power. Qual Health Res.

[11] Braun V, Clarke V (2022). Thematic analysis: a practical guide.

[12] Eaton G, Tierney S, Wong G (2022). Understanding the roles and work of paramedics in primary care: a national cross-sectional survey. BMJ Open.

[13] O’Sullivan JW, Stevens S, Hobbs FDR (2018). Temporal trends in use of tests in UK primary care, 2000-15: retrospective analysis of 250 million tests. BMJ.

[14] Watson J, Whiting PF, Salisbury C (2022). Blood tests in primary care: a qualitative study of communication and decision-making between doctors and patients. Health Expect.

[15] Gill PJ, Hislop J, Mant D, Harnden A (2012). General practitioners’ views on quality markers for children in UK primary care: a qualitative study. BMC Fam Pract.

[16] Watson J, de Salis I, Hamilton W, Salisbury C (2016). ‘I’m fishing really’ — inflammatory marker testing in primary care: a qualitative study. Br J Gen Pract.

[17] Hoffmann TC, Del Mar C (2015). Patients’ expectations of the benefits and harms of treatments, screening, and tests: a systematic review. JAMA Intern Med.

[18] Hoffmann TC, Del Mar C (2017). Clinicians’ expectations of the benefits and harms of treatments, screening, and tests: a systematic review. JAMA Intern Med.

[19] Walker GJ, Chanchlani N, Thomas A (2020). Primary care faecal calprotectin testing in children with suspected inflammatory bowel disease: a diagnostic accuracy study. Arch Dis Child.

[20] Wang Z, Pianosi PT, Keogh KA (2018). The diagnostic accuracy of fractional exhaled nitric oxide testing in asthma: a systematic review and meta-analyses. Mayo Clin Proc.

[21] Tang S, Xie Y, Yuan C (2019). Fractional exhaled nitric oxide for the diagnosis of childhood asthma: a systematic review and meta-analysis. Clin Rev Allergy Immunol.

